# Oral Streptococci Utilize a Siglec-Like Domain of Serine-Rich Repeat Adhesins to Preferentially Target Platelet Sialoglycans in Human Blood

**DOI:** 10.1371/journal.ppat.1004540

**Published:** 2014-12-04

**Authors:** Lingquan Deng, Barbara A. Bensing, Supaporn Thamadilok, Hai Yu, Kam Lau, Xi Chen, Stefan Ruhl, Paul M. Sullam, Ajit Varki

**Affiliations:** 1 Glycobiology Research and Training Center, Departments of Medicine and Cellular & Molecular Medicine, University of California, San Diego, San Diego, California, United States of America; 2 Department of Medicine, San Francisco Veterans Affairs Medical Center and the University of California, San Francisco, San Francisco, California, United States of America; 3 Department of Oral Biology, School of Dental Medicine, University at Buffalo, Buffalo, New York, United States of America; 4 Department of Chemistry, University of California, Davis, Davis, California, United States of America; The University of Texas Health Science Center at San Antonio, United States of America

## Abstract

Damaged cardiac valves attract blood-borne bacteria, and infective endocarditis is often caused by viridans group streptococci. While such bacteria use multiple adhesins to maintain their normal oral commensal state, recognition of platelet sialoglycans provides an intermediary for binding to damaged valvular endocardium. We use a customized sialoglycan microarray to explore the varied binding properties of phylogenetically related serine-rich repeat adhesins, the GspB, Hsa, and SrpA homologs from *Streptococcus gordonii* and *Streptococcus sanguinis* species, which belong to a highly conserved family of glycoproteins that contribute to virulence for a broad range of Gram-positive pathogens. Binding profiles of recombinant soluble homologs containing novel sialic acid-recognizing Siglec-like domains correlate well with binding of corresponding whole bacteria to arrays. These bacteria show multiple modes of glycan, protein, or divalent cation-dependent binding to synthetic glycoconjugates and isolated glycoproteins in vitro. However, endogenous asialoglycan-recognizing clearance receptors are known to ensure that only fully sialylated glycans dominate in the endovascular system, wherein we find these particular streptococci become primarily dependent on their Siglec-like adhesins for glycan-mediated recognition events. Remarkably, despite an excess of alternate sialoglycan ligands in cellular and soluble blood components, these adhesins selectively target intact bacteria to sialylated ligands on platelets, within human whole blood. These preferred interactions are inhibited by corresponding recombinant soluble adhesins, which also preferentially recognize platelets. Our data indicate that circulating platelets may act as inadvertent Trojan horse carriers of oral streptococci to the site of damaged endocardium, and provide an explanation why it is that among innumerable microbes that gain occasional access to the bloodstream, certain viridans group streptococci have a selective advantage in colonizing damaged cardiac valves and cause infective endocarditis.

## Author Summary

Bacterial infective endocarditis remains a disease with considerable morbidity and mortality. Of the numerous bacteria that can enter the bloodstream, certain oral commensal viridans group streptococci are among the major causative organisms of endocarditis. However, mechanisms underlying this selectivity are incompletely understood. Interactions between adhesins of such bacteria and human platelet sialoglycans are believed to play an important role in this selectivity, by facilitating bacterial adherence to damaged heart valves. Nevertheless, the molecular requirements for these interactions are not fully explored. Particularly, it is unclear whether selective targeting of platelets by these bacteria actually occurs in fluid human whole blood, an environment where numerous potential sialoglycan competitors exist. In the present work, we have addressed these important issues. We characterize in detail the glycan-binding spectra of a series of serine-rich repeat adhesins of oral streptococci. For the first time, we demonstrate that oral streptococci can indeed selectively target platelets in whole human blood. As a proof of concept, we also show that soluble recombinant bacterial adhesin binding region proteins can block the preferred platelet-bacterial interactions in whole blood. The knowledge gained from this study may help the development of novel preventive or therapeutic approaches against infective endocarditis.

## Introduction

Infective endocarditis (IE) remains a disease with considerable morbidity and mortality [1], [2]. Of the numerous bacteria that have the opportunity to enter the bloodstream, three major genera of Gram-positive pathogens (streptococci, staphylococci and enterococci) dominate in IE. Streptococci and staphylococci account for 80% of IE cases [3], and viridans group streptococci, including *Streptococcus gordonii* and *Streptococcus sanguinis*, make up to half of such cases [4]. Unlike the intrinsically virulent staphylococci, streptococci causing IE are commensal species that normally reside in the oral cavity and show relatively weak or no pathogenicity. However, they have the potential to cause life-threatening IE when they enter the bloodstream through lesions in the oral epithelia or after dental procedures [5]. The question arises as to why these particular organisms have such a selective advantage in causing IE. Answers to this question are of importance in preventing and treating this serious disease.

The pathogenesis of IE is complex but bacterial adherence to damaged heart valves is a required event [1], [6]. Platelets appear to play a crucial role in pathogenesis, as the streptococci associated with IE can bind to platelet components to initiate adherence to damaged human heart valves [7]. Several streptococcal adhesins are known to bind human platelets [8]–[15]. Among the best characterized are GspB and Hsa of *S. gordonii* and SrpA of *S. sanguinis*, all belonging to a conserved family of serine-rich repeat (SRR) glycoproteins [13]–[15]. The SRR glycoproteins and corresponding specialized secretion systems are being defined in a growing number of pathogens, indicating their important roles in the behavior of Gram-positive organisms [16], [17]. However, we are only beginning to understand the functions of this interesting family of proteins.

All human cell surfaces are covered by a dense and complex layer of glycans [18]–[20]. Glycan-mediated host-pathogen interactions are involved in various human disease processes [21]. Sialic acids (Sias) are a diverse group of α-keto acids with a shared nine-carbon backbone [22], [23]. Given their presence at terminal positions of cell surface and secreted glycoconjugates, they mediate or modulate various biological interactions [24], [25]. Interestingly, GspB, Hsa and SrpA are all Sia-binding adhesins. GspB, the SRR adhesin from *S. gordonii* strain M99, was recently found to contain a subdomain in its binding region (BR) [26], with the topology and strand inserts similar to the V-set Ig-like fold adopted by mammalian sialic acid binding immunoglobulin-like lectins (Siglecs) [27]. Each of the other two homologous SRR adhesins, Hsa from *S. gordonii* strain DL1 and SrpA from *S. sanguinis* strain SK36, also contains a Siglec-like subdomain in their BRs [26].

Taken together, existing data suggests that the Sia-binding capabilities of GspB, Hsa and SrpA are conferred by their Siglec-like modules, and that such binding assists interactions with platelets. This surface property may help targeting the bacteria to the coagulum made of platelets and other components on damaged cardiac valves and act as a contributory factor in the pathogenesis of IE. However, detailed characterization of cognate ligands of such adhesins is lacking. It is also not known whether these bacteria could selectively target platelets in fluid human whole blood, a process that is under-explored but may contribute to the pathogenesis of IE [1]. This question is of particular interest also because the endovascular system is an environment where numerous potential sialoglycan competitors exist [28]. Such selectivity would thus play an essential role for the successful delivery and adhesion of bacteria to damaged valvular endocardium, and provide an explanation to the selective advantage of certain types of bacteria in causing IE.

Here we utilize a number of complementary techniques and novel assays to study the role of the Siglec-like domain-containing SRR-adhesins in bacterial recognition of host sialoglycans. We explore mechanisms of these bacterial interactions with saliva and in whole blood. Our finding extends previous understanding of the mechanisms of infective endocarditis. The novel insights gained help us to better understand the contribution of Sia-binding adhesins in the pathogenesis of IE. In particular, we address questions including the following: 1) What are the detailed sialoglycan-binding characteristics of GspB-BR, Hsa-BR, and SrpA-BR?; 2) Are the Siglec-like BRs of these adhesins responsible for whole bacterial recognition of sialoglycans?; 3) Are Siglec-like domain-containing SRR adhesins common among oral streptococci, and is there a good phylogeny-function relationship?; 4) How do these oral streptococci interact with saliva from the oral cavity, and with blood components from the bloodstream?; and 5) Can these bacteria preferentially recognize platelets in the setting of whole human blood via Sia-adhesin interactions, despite numerous potential sialoglycoprotein competitors from plasma and surfaces of other blood cells?

In addressing the last issue, we have developed a novel whole blood-whole bacterium flow cytometry assay that is most relevant to bona fide human conditions. For the first time, we have demonstrated that oral streptococci can indeed selectively target platelets in human whole blood. It appears that circulating platelets may act as inadvertent Trojan horse carriers of oral streptococci to the site of damaged endocardium. By serendipity, certain sialoglycan-binding properties that facilitate normal oral commensalism seem to have set these bacteria up to be inadvertent, yet highly specific causative agents of endocarditis, via platelet intermediaries. As a proof of concept, we have also shown that soluble recombinant bacterial adhesin binding region proteins can block the preferred platelet-bacterial interactions in whole blood. The knowledge gained may contribute to the development of novel preventive or therapeutic approaches against infective endocarditis.

## Results

### Glycan microarray analyses show varied Sia-binding specificities of streptococcal adhesins containing a Siglec-like domain

**Figure 1:**
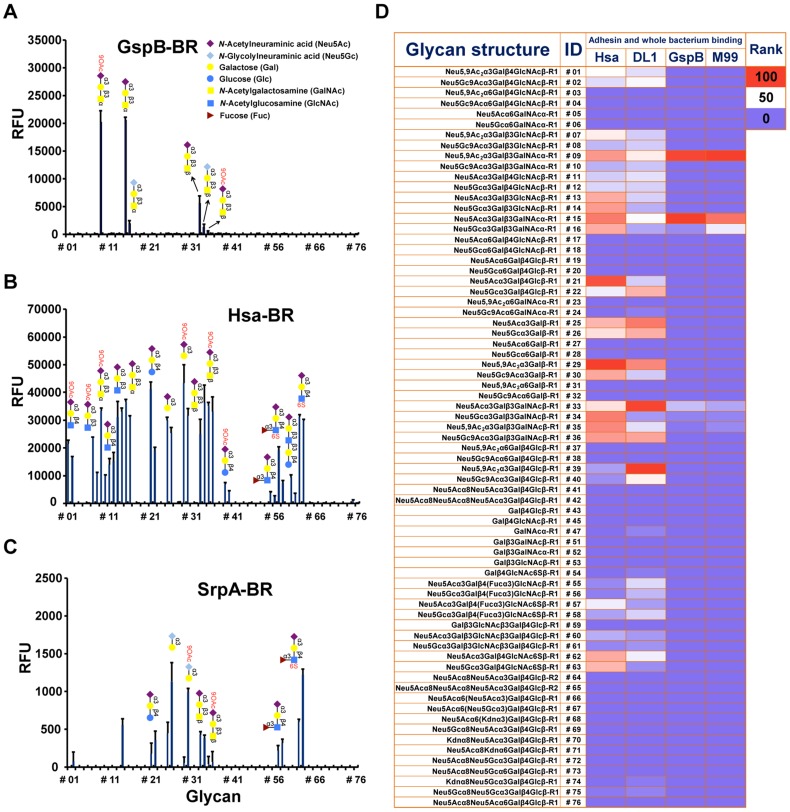
Sialoglycan microarray analysis of binding specificities of adhesin-BRs and corresponding whole bacteria. (A–C) Binding of the adhesin-BR proteins at 50 nM is presented. (n = 4, SD). (D) Adhesin-BR proteins and Cy3-labeled whole bacteria were assayed using the same sialoglycan microarrays. Heat map was generated using the method as previously described [29]. R1 and R2 represent two different spacers.

Glutathione *S*-transferase (GST)-tagged BRs of GspB, Hsa, and SrpA (see Table S1 for details), were tested for binding to various sialoglycans, using a recently developed slide microarray. This unique sialoglycan microarray presents over 70 synthetically recreated naturally-occurring oligosaccharide structures with diverse sialic acid forms, glycosidic linkages, and underlying glycans, representing the broadest range of such targets available to date [23], [29]. All three proteins exclusively bound to glycans terminated with sialic acids but not non-sialylated ones (Figure 1A–1D). However, they also showed distinct specificities.

GspB-BR interacted with a very narrow spectrum of sialoglycans, primarily Neu5Acα2-3Galβ1-3GalNAcα1- (Glycan #15, the sialyl T antigen, sTa) and related structures (Figure 1A and 1D). 9-*O*-Acetylation of the Neu5Ac moiety of sTa (Glycan #9, compared to #15) did not influence binding significantly. Sulfation of underlying glycans also showed no effect (Glycans #57, 58 and 62, 63, compared to #55, 56, and 11, 12, respectively). In contrast, Hsa-BR bound a broad range of Sias with different underlying glycans (Figure 1B and 1D). Conspicuously, it recognized all α2-3-linked Sias but not any α2-6- or α2-8-linked ones. This finding expands on previous studies, which showed that Hsa could bind α2-3-linked Sias with three different underlying glycans, specifically, sTa, Neu5Acα2-3Galβ1-4GlcNAcβ1- (3′SLn) and Neu5Acα2-3Galβ1-4Glcβ1- (3′SL) structures [30], [31]. Our microarray displays Sias with over 10 different underlying glycans (Table S2), and it appears that every α2-3-linked Sia present on the array could be bound by Hsa-BR (Glycans #1, 2, 7-16, 21, 22, 25, 26, 29, 30, 33-36, 39, 40, 55-58, and 60-63) (Figure 1B and 1D). Similar to GspB-BR, 9-*O*-acetylation on Sia did not block Hsa-BR binding. In contrast to GspB-BR, Hsa-BR could not only recognize tri- and oligo-saccharide Sias, but also di-saccharide Sias (Glycans #25, 26, 29, 30). Sulfation significantly increased binding (Glycans #57, 58 and 62, 63, compared to #55, 56, and 11, 12, respectively). Notably, SrpA-BR recognized sialoglycans in a manner resembling Hsa-BR more than GspB-BR (Figure 1C), showing binding to both di-saccharide and tri-/oligo-saccharide Sias. Sulfation also increased binding. Moreover, we tested the maltose-binding protein (MBP)-tagged Hsa-BR, which showed the same binding characteristics as GST-tagged Hsa-BR (Figure S1), indicating that tagging methods did not alter adhesin binding.

### Whole bacteria bind to glycan microarrays in patterns similar to those of their adhesin-BR fusion proteins

To test whether the SRR adhesin-BR fusion proteins faithfully represent the binding properties of the corresponding whole organisms, we assessed direct binding of strains M99, DL1, and SK36 to the same microarray. To confirm that binding was due to expression of the adhesin, we also tested isogenic variants of each strain, in which the gene encoding the SRR glycoprotein had been deleted (M99Δ*gspB*, DL1Δ*hsa*, and SK36Δ*srpA*). Prior glycan microarray studies using whole bacteria were characterized by high background noise or no success at all (http://www.functionalglycomics.org/glycomics/publicdata/selectedScreens.jsp). However, our current efforts at optimization yielded good signal-to-noise ratios (Figure S2). Notably, DL1 and M99 showed nearly identical binding profiles as their respective adhesins, Hsa and GspB, on the array (Figure 1D). In keeping with the relatively low binding of SrpA to the arrayed Sias (see mean relative fluorescence intensities in Figure 1A–1C), the corresponding bacteria SK36 showed minimal binding. Importantly, all adhesin-deficient mutant strains showed no binding to the sialosides on the microarrays. Thus, whole bacterial binding to the immobilized sialoglycans was SRR adhesin-dependent.

### Phylogeny of binding regions of Siglec-like domain-containing adhesins from selected strains corroborates binding result

**Figure 2:**
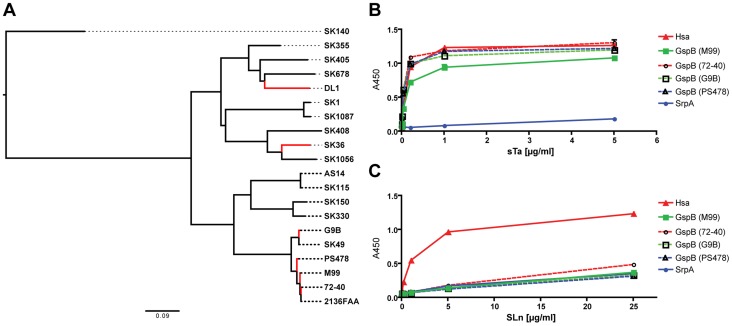
Phylogeny of SRR glycoprotein BRs from a representative number of *S. sanguinis* and *S. gordonii* strains, and glycan binding to immobilized adhesin-BRs. (A) ClustalW2 was used for both the sequence alignment and the neighbor-joining tree reconstruction. Corresponding SRR adhesin-BR sequence accession numbers are provided in Table S3. (B) PAA-supported sTa binds to both the Hsa-BR and BRs of the four GspB-orthologues. (C) PAA-SLn only binds to Hsa-BR. (n = 6, SEM).

The SRR glycoproteins comprise a large family of adhesins in Gram-positive bacteria [16]. To determine the prevalence of Siglec-like domain-containing adhesins in the family, we searched public databases and gathered additional sequence data from our lab collection (strains PS478, 72-40, and G9B). Seventeen additional predicted SRR homologs of GspB, Hsa, and SrpA were identified based on their Siglec-like domain-containing BR sequences (Figure 2A). While GspB clustered with five other SRR proteins, Hsa and SrpA appeared in a different branch, and were more closely related to each other than to GspB. The phylogenetic relationship agreed with their functional relationships in terms of ligand repertoire (Figure 1A–1C). To further demonstrate this phylogeny-function relationship, we produced additional GST-tagged adhesin-BR proteins (GspB_PS478_BR, GspB_72-40_BR, and GspB_G9B_BR), which differ from GspB_M99_BR by only a few amino acid residues (Figure S3). We then labeled the corresponding whole bacteria (strains PS478, 72-40, and G9B), and tested the organisms together with their adhesin-BRs on the same sialoglycan microarray. The new adhesin-BRs displayed binding properties that were nearly identical to those of GspB_M99_BR (Figure S4). The strains also showed binding patterns nearly identical to that of their adhesins, as well as to strain M99, whereas an adhesin-deficient mutant of 72-40 (PS1070) [32] showed no binding to any sialoglycan. Moreover, the glycan-binding properties of all above-mentioned adhesin-BR fusion proteins were confirmed by a conventional enzyme-linked lectin assay [26], using two model glycans, sTa and 3′SLn (Figure 2B and 2C).

Taken together, the data show that the Siglec-like domain-containing bacterial adhesins are prevalent in *S. gordonii* and *S. sanguinis* species, which are among the most common causative agents of IE. The data also indicate that GspB, Hsa and SrpA studied in the present work are representatives of many Sia-binding SRR proteins. The results and methods from this work should thus be broadly applicable.

### Bacteria show multiple modes of binding to human saliva, purified glycoproteins and related glycoconjugates

Streptococci normally reside in the oral cavity, where they use multiple adhesins for commensal interactions [33], and only a subset of strains are known to become opportunistic pathogens in the endovascular environment, causing diseases such as IE. We thus wanted to probe the broader range of adhesins on DL1, M99 and SK36, asking if they use the same adhesins in the oral cavity for colonization and in the bloodstream. We used a dot blot assay to assess bacterial binding to whole human saliva, salivary ductal secretions, isolated and purified glycoproteins, and related glycoconjugates. In addition, we examined whether binding required divalent cations.

**Figure 3:**
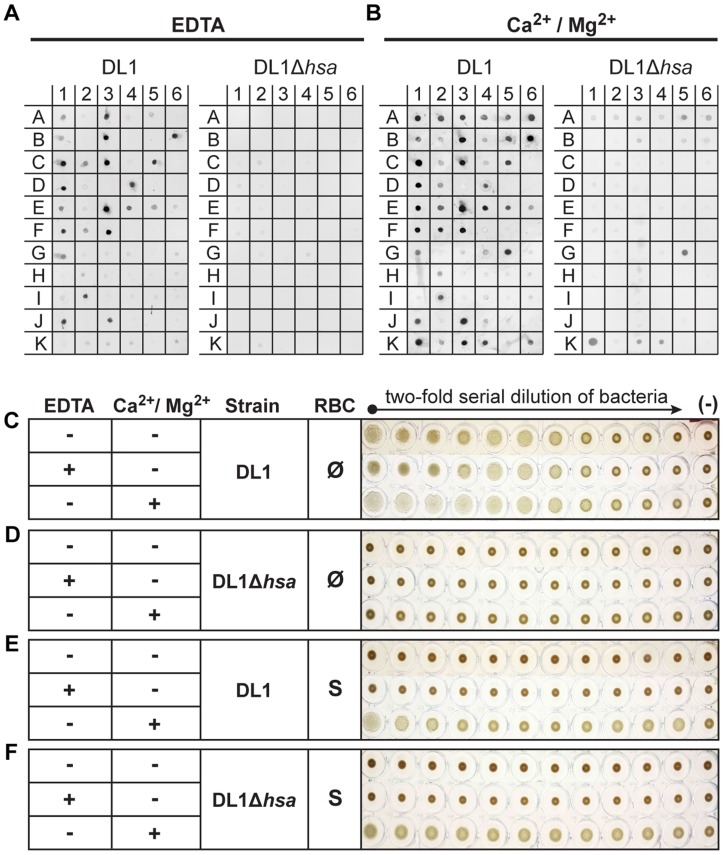
Bacterial binding to saliva and glycoconjugates, and bacterium-mediated hemagglutination. (A and B) Binding of DL1 vs. DL1Δ*hsa* against immobilized whole human saliva, salivary ductal secretions, isolated and purified glycoproteins and related glycoconjugates, was tested by a dot blot assay. The binding was studied in the presence of EDTA (A) or divalent cations (B). (C–F) Bacterium-mediated hemagglutination using human RBCs and two-fold serial diluted bacterial suspensions. (C and D) DL1- and DL1Δ*hsa*-mediated hemagglutination using non-sialidase treated (Ø) RBCs. (E and F) DL1- and DL1Δ*hsa*-mediated hemagglutination using sialidase treated (S) RBCs.

Under divalent cation-chelating conditions (+EDTA) (Figure 3A, see substrate info in Figure S5), wild type DL1 bound saliva samples A1, A3, and A5, but not de-sialylated samples A2, A4 and A6. Similarly, DL1 interacted with various sialylated glycoproteins but showed much reduced or no binding to corresponding desialylated glycoproteins. These included, for example, salivary mucins MUC5B (B1) and MUC7 (B3). When the adhesin-deficient strain DL1Δ*hsa* was used under the same conditions in the bacterial overlay, binding was not seen. For M99 and SK36, the Sia-dependent interactions were also absent when corresponding adhesin-deficient strains were used (Figure S5). These data show that the Siglec-like SRR adhesins are involved in bacterium-saliva and various sialoglycoprotein interactions, and confirm that they interact with their Sia-ligands in a Ca^2+^/Mg^2+^ independent manner [26].

In contrast, the binding profiles changed considerably in the presence of Ca^2+^/Mg^2+^ (Figure 3B). Additional binding activities that did not involve Sias were observed and those interactions largely obscured the Sia-dependent patterns seen in EDTA. Most conspicuously, DL1 bound to *N*-acetylgalactosamine (GalNAc)-terminated structures (G5, K1, K3 and K4), and these interactions were still present with the Hsa-deficient mutant. This fits previous findings [34], indicating that lectin-like adhesins apart from the Sia-binding ones are also expressed on such oral streptococci. Similarly, M99 bound to terminal β-galactose (Gal) glycan structures (I6, and K6), while SK36 also interacted with GalNAc-terminated structures, as was seen with DL1 (G5, K1, K3 and K4) (Figure S5).

Taken together, the data indicate that these bacteria express additional adhesins beyond Sia-binding ones. These multiple adhesins likely evolved to interact with salivary and oral mucosal glycoproteins and/or other glyco-epitopes, e.g. the ones present in Gal/GalNAc receptor polysaccharides on coaggregating bacterial strains [35], which could be essential for their biofilm formation and colonization in the oral cavity. Moreover, the asialoglycan-binding adhesins require divalent cations for action, unlike the Sia-binding SRR-adhesins.

### Streptococcal strains primarily depend on Sia-binding adhesins to hemagglutinate red blood cells in vitro

When oral streptococci enter the bloodstream, they encounter a drastically different environment, with regard to terminal glycan sequences available for adhesin binding. Unlike the case in the oral cavity, terminal Gal and GalNAc residues are not tolerated on plasma- or blood cell surface glycoconjugates, as they are immediately recognized for clearance by receptors on hepatocytes or macrophages [36], [37]. Instead, the endovascular system is an environment where numerous sialoglycans exist [28]. Thus, the Sia-binding SRR adhesins should theoretically become the more prominent determinants of glycan-mediated binding in the bloodstream. To test this hypothesis we probed interactions of DL1 and DL1Δ*hsa* with human RBCs in traditional hemagglutination assays, in the presence or absence of EDTA (Figure 3C–3F). Hemagglutination was comparable under all conditions when untreated RBCs were used (Figure 3C, all three rows), i.e., independent of divalent cations. Moreover, no hemagglutination was observed with DL1Δ*hsa* under any condition (Figure 3D, all three rows). Thus, GalNAc- or Gal-binding adhesins do not contribute significantly to bacterial hemagglutination, which is predominantly mediated by Sia-recognition.

In contrast, when the RBCs were sialidase-treated and underlying glycans were exposed, DL1 bound avidly to such RBCs in the presence of Ca^2+^/Mg^2+^ (Figure 3E, third row). Furthermore, this was independent of the Sia-binding adhesin Hsa (Figure 3F, third row). These hemagglutination reactions were most likely mediated by GalNAc- and/or Gal-binding adhesins expressed on DL1, when their ligands on RBCs became exposed by sialidase treatment, and in the presence of divalent cations. Indeed, neither DL1 nor DL1Δ*hsa* showed any hemagglutination of sialidase-treated RBCs when Ca^2+^/Mg^2+^ were absent (Figure 3E and 3F, first two rows).

Taken together, the results fit the finding that normal RBCs are primarily covered by sialoglycans without many exposed GalNAc or Gal residues [38]. Although DL1 possesses both the Sia-binding adhesin and other adhesins including other types of lectin-like adhesins, the bacterium depends primarily on the former to bind RBCs. In the oral cavity, where these streptococci normally reside, they likely utilize many kinds of adhesins evolved for colonization [34], [39]. However, when they enter the blood stream, an environment where Sias dominate the terminal glycome, they become primarily dependent on their Sia-reactive SRR adhesins to mediate binding.

### Comparative binding of isogenic pairs of bacteria to human blood components confirm the critical role of SRR adhesins and host sialic acids

**Figure 4:**
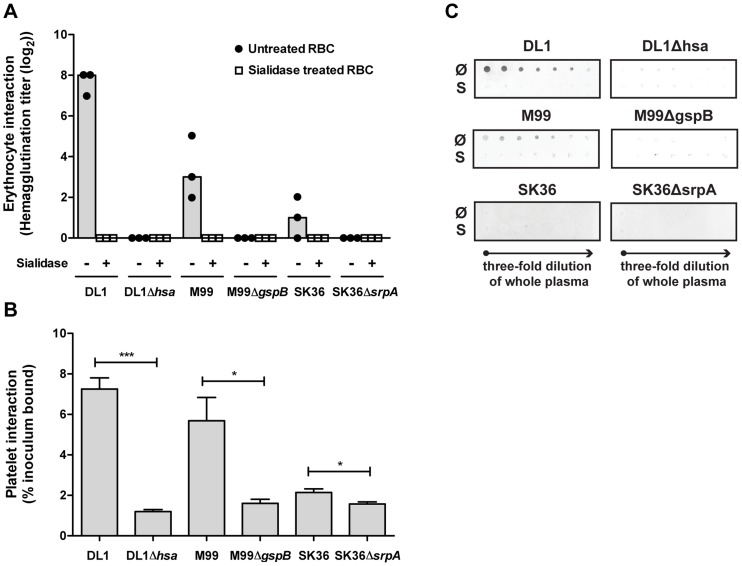
Bacterial interactions with separated erythrocytes, platelets, and whole human plasma. (A) Wild type bacteria only hemagglutinate non-sialidase treated RBCs. Mutant strains do not show any hemagglutination. Bars represent median values. (n = 3). (B) Bacterial adhesion to fixed, immobilized platelets. (n = 6, SEM). (C) Whole plasma samples were immobilized on dot blot in three-fold serial dilutions starting from the original concentration in whole blood. Each strain was tested to interact with either non-sialidase treated (Ø) or sialidase treated whole plasma (S). *P<0.05, ***P<0.001.

We then systematically compared binding of the three isogenic pairs of strains to blood cells, including RBCs and platelets (Figure 4A and 4B). Hemagglutination assays confirmed that the sialic acid-mediated interactions of DL1, M99 and SK36 with human RBCs were dependent on their respective SRR adhesins, Hsa, GspB and SrpA (Figure 4A). An immobilized platelet adhesion assay showed similar trends among the three strains (Figure 4B). Notably, the SRR adhesin-deficient mutant strains displayed residual binding to platelets, indicating that other adhesins expressed on such strains could contribute to the platelet-bacterial interactions. We also tested bacterial binding to immobilized whole human plasma glycoproteins by dot blot (Figure 4C). Both DL1 and M99 showed clear Sia- and Hsa-dependent binding to whole plasma. In contrast, SK36 showed minimal binding to whole plasma under the conditions tested.

### A novel whole bacterium-whole blood flow cytometry (WBWB-FC) assay shows that oral streptococci preferentially adhere to platelets in intact human whole blood

Although binding of the different strains to separate human blood components has been studied by us and others, evaluation of such binding in human whole blood has not been addressed to date. However, the latter approach is most biomedically relevant. Bacteria entering the bloodstream would encounter ∼2 mM concentration of competing sialoglycoproteins in the plasma [40] and over 100-fold larger total RBC surface area than that of platelets (average single RBC surface area: 2S = 2×πr^2^ = 2×3.14×(4 µm)^2^, and average RBC count: 4.40–6.00×10^6^/µL; average single platelet surface area: 2S = 2×πr^2^ = 2×3.14×(1.5 µm)^2^, and average platelet count: 0.14–0.40×10^6^/µL). Thus the question arises as to whether these bacteria can preferentially detect platelets under such conditions.

**Figure 5:**
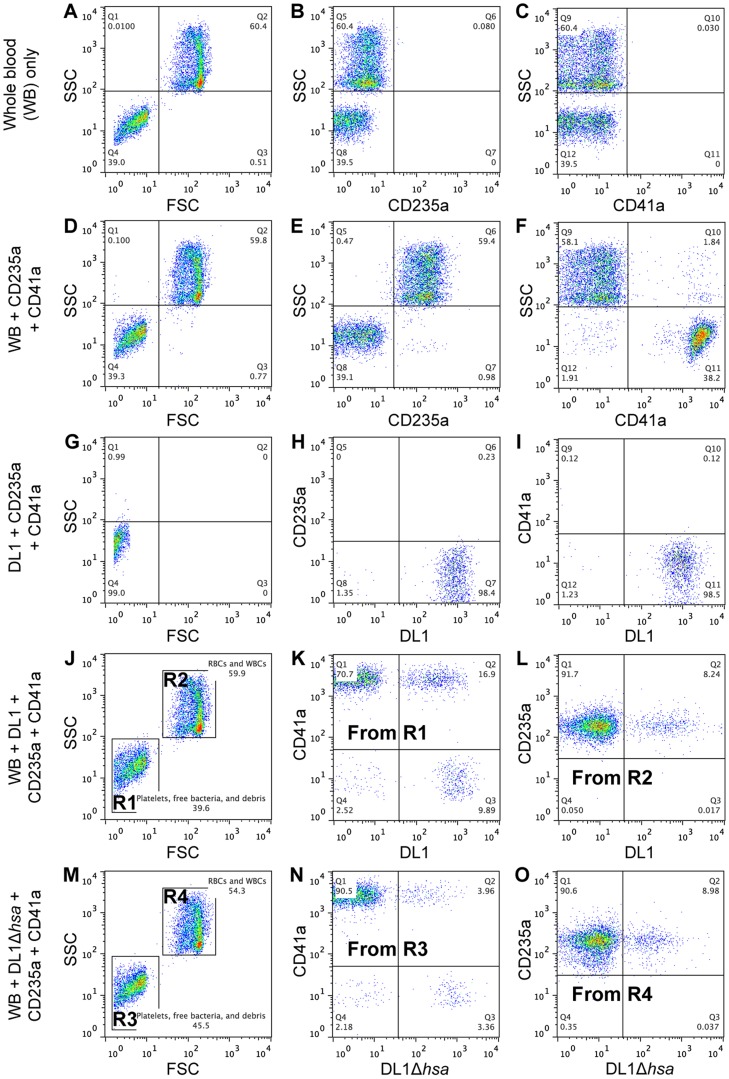
Representative flow cytometry profiles showing preferential platelet-bacterial adherence in intact whole human blood. (A–C) Whole blood (WB) in PBS. (D–F) Two antibodies, CD235a-FITC and CD41a-APC, were added to WB. (G–I) DL1-Cy3 incubated with CD235a-FITC and CD41a-APC: (G) DL1-Cy3 also appears in Q4 in the FSC/SSC plot, the same as platelets. (J–L) DL1-Cy3, CD235a-FITC and CD41a-APC were added to WB: (J) Platelets/bacteria are gated as R1 and RBCs/WBCs as R2. (K) Events from gate R1. (L) Events from gate R2. Bacteria preferentially recognize platelets compared to RBCs. (M–O) DL1Δ*hsa*-Cy3, CD235a-FITC and CD41a-APC were added to WB: (N) Events from gate R3, and DL1Δ*hsa*-Cy3 bound platelets are drastically reduced compared to DL1-Cy3 bound platelets. (O) Events from gate R4. Blood samples from multiple donors were tested and consistent results were obtained.

To address this question we developed a novel whole bacterium-whole blood flow cytometry (WBWB-FC) assay, in which fluorochrome-labeled bacteria and antibodies were mixed into fresh anti-coagulated human blood, allowed to interact for short time, and then rapidly diluted before immediate analysis by flow cytometry. Two distinct populations of cells were seen when the mixture was analyzed by forward (FSC) and side scatter (SSC) (Figure 5A, 5D, 5J and 5M). Erythrocytes were identified by the expression of glycophorin A (CD235a) (Figure 5E), and platelets by GPIIb (CD41a) (Figure 5F). Minimum non-specific interactions were seen as a result of careful selection of antibodies and optimization of assay conditions. Bacteria showed no interaction with the antibodies used (Figure 5G–5I). When wild type DL1 was added in whole human blood, it preferentially recognized platelets as compared with RBCs (Figure 5K and 5L, note the percentage values of Q2/Q1). When DL1Δ*hsa* was used, the preferred platelet-bacterial interaction was lost (Figure 5N, compare to 5K and 5O). The M99 and SK36 isogenic pairs gave similar results, albeit lower extent of preferential platelet binding by wild type M99 or SK36 was observed compared to by DL1.

**Figure 6:**
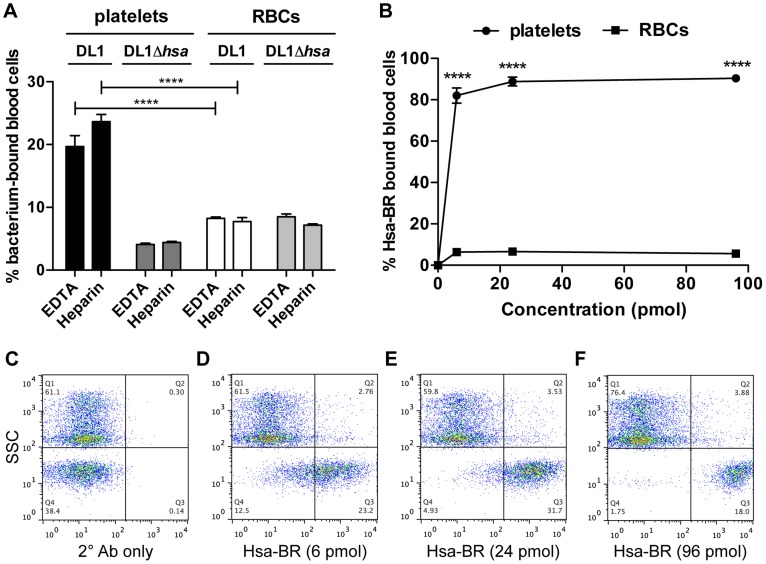
Divalent cation effect on bacterial binding and preferential recognition of platelets over RBCs by Hsa-BR in whole blood. (A) DL1 and DL1Δ*hsa* binding to either platelets or RBCs were comparable in EDTA- or heparin-anticoagulated whole blood. Bar values = 100×[number of bacterium-bound platelets (or RBCs)/total number of platelets (or RBCs)]. (n = 3, SEM). (B) Quantification and comparison of Hsa-BR binding to platelets and RBCs in whole blood. Bar values = 100×[number of HsaBR-bound platelets (or RBCs)/total number of platelets (or RBCs)]. (n = 6, SEM). (C) Q1 shows mainly RBCs, and Q4 platelets. Only 2° antibody, anti-GST-APC, alone was added in whole blood. (D–F) 6 to 96 pmol of Hsa-BR was added to whole blood, followed by anti-GST-APC. ****P<0.0001.

We next determined the effect of divalent cations on whole bacterium-whole blood bindings (Figure 6A). Interactions were largely comparable in either EDTA or heparin anti-coagulated blood, indicating that divalent cations did not influence binding events much. In addition, an anti-P-selectin antibody was used to monitor platelet activation during the course of whole blood-bacterial binding experiments. Minimum activation was observed, indicating that these bacteria interacted with platelets in their resting state (Figure S6).

Thus, for the first time, we have demonstrated that the viridans group streptococcal strains can utilize their Siglec-like domain-containing SRR adhesins to preferentially bind platelets in the setting of human whole blood, despite numerous potential binding competitors, e.g., plasma- and other blood cell surface sialoglycoproteins. Divalent cations in whole blood also showed minimal effect on these bacterial interactions with blood cells, supporting the dominant roles of Sia-binding adhesins for streptococcal recognitions in the blood stream.

### Bacterial adhesin BR proteins preferentially recognize platelets in whole blood similar to corresponding whole bacteria

To pursue future studies in this area it would be important to know if the recombinant adhesin-BR proteins can also preferentially detect platelets in the setting of human whole blood. As a proof of concept, we tested Hsa-BR. Notably, it indeed clearly displayed a strong preference to bind human platelets compared to RBCs (Figure 6B). Also, it increasingly adhered to platelets when more protein was used (Figure 6D–6F and Figure S7). As a control, secondary antibody alone did not distinguish platelets from RBCs (Figure 6C). These data indicate that sialic acid ligand density, accessibility, and/or particular sialoglycan presentation [41] on platelets play an essential role in mediating the preferred platelet-bacterial binding.

### Adhesin BR proteins can block favored bacterium-platelet interactions in human whole blood

**Figure 7:**
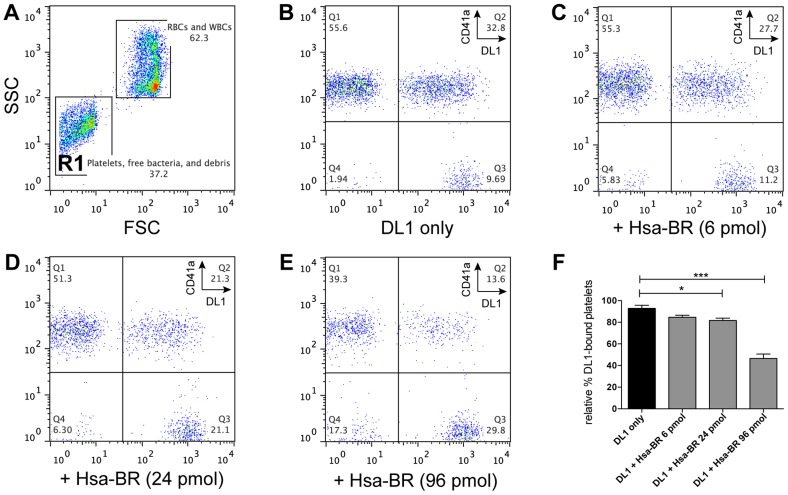
Blocking of DL1-platelet binding by Hsa-BR in whole human blood. (A) Representative FSC/SSC plot showing whole blood with added DL1-Cy3. Platelets and bacteria are gated as R1. (B–E) are gated from R1: (B) Whole blood with DL1-Cy3 and CD41a-APC. Non-DL1-bound platelets appear in Q1 and DL1-bound in Q2, while free DL1-Cy3 in Q3. (C–E) Whole blood with different concentrations of Hsa-BR added first, followed by DL1-Cy3 and CD41a-APC. Increasing amount of Hsa-BR results in decreasing count of DL1-bound platelets (Q2 percentages) and increasing numbers of free bacteria (Q3 percentages). (F) Quantification of Hsa-BR blocking of DL1 binding to platelets in whole blood. The percentage values of DL1-bound platelets were normalized, with the highest single value being 100%. (n = 6, SEM). *P<0.05, ***P<0.001.

Based on the above data, we further hypothesized the recombinant adhesin-BR proteins would be capable of inhibiting the preferred platelet-bacterial interaction in human whole blood. We thus tested Hsa-BR in inhibiting DL1-platelet binding. It was found that the favorable DL1-platelet interaction was gradually inhibited by increasing concentrations of added soluble recombinant Hsa-BR protein (Figure 7). In keeping with this blocking of platelet-bacterial interactions, increasing numbers of un-bound bacteria were detected with increasing amounts of recombinant Hsa-BR added (Figure 7C–7E, quadrant 3). Furthermore, the adhesin-blocking effect was verified by platelet adhesion assay using washed and immobilized platelets (Figure S8).

## Discussion

The incidence of and mortality from IE has not reduced over the past three decades, despite significant improvements seen in most other diseases during the same period of time [4]. The incidence of IE is about 1.4–12.7 per 100,000 person-years [1], and mean in-hospital mortality is as high as 20% and one-year mortality up to 40% [4]. Without treatment, IE is often lethal. In short, IE remains an important medical and socioeconomic burden to date.

Bacterial adhesins play critical roles in host-microbe interactions, mediating host signaling events and affecting bacterial uptake or invasion [42]. Sialic acid-binding adhesins of viridans group streptococci are among the first identified lectin-like adhesins [43], [44]. This is not surprising considering the ubiquity of sialic acids on host cell surfaces and in mucosal secretions, and their usual occurrence at terminal positions of various glycoproteins and glycolipids [25]. Notably, 70%–90% of streptococcal strains from a collection of *S. sanguinis*, *S. gordonii*, and *S. oralis* species were reported to express sialic acid-reactive adhesins, unlike the other streptococcal species such as the *S. anginosus* [45]. This is in intriguing coincidence with independent findings that *S. sanguinis*, *S. gordonii*, and *S. oralis* are among the leading causative organisms of streptococcal IE [46]. Taken together, the Sia-adhesin mechanism we have elaborated in the current study might play a much more prevalent role in the pathogenesis of IE than currently recognized. Of course, such a mechanism may not be restricted to SRR type adhesins that we emphasize in the present work.

On a broader scale, SRR glycoproteins constitute an important family of conserved cell surface proteins associated with Gram-positive pathogenesis. Among the important questions arising, it is imperative to achieve full characterization of cognate ligands for these adhesins [16]. In this regard, glycan microarrays serve as a valuable tool. It allows simultaneous screening of hundreds of glycan-protein interactions in a high-throughput manner and only requires minimal materials. For the first time, we characterize in detail the glycan-binding spectra of a series of serine-rich repeat adhesins of oral streptococci. This is a highly conserved family of glycoproteins that contribute to virulence for a broad range of Gram-positive pathogens [16]. The information may help us to understand tissue and site tropism of the streptococcal adhesion within the oral cavity and elsewhere.

Useful glycoanalytical probes are also identified. The bacterial adhesin Hsa-BR fusion protein shows great promise in replacing plant lectins MAL-I and MAL-II as a broad-range probe for α2-3-linked sialic acids. For instance, to our knowledge, there was not a good lectin for glycan microarray quality control to probe all α2-3-linked sialic acids. Hsa-BR will thus prove useful in such efforts as well as in other applications including cell and tissue staining. It is also gratifying to have achieved successful whole bacterial binding onto sialoglycan microarrays, which enables direct comparison of glycan-binding properties between whole bacteria and their corresponding adhesins. This approach not only confirms that adhesin-BRs faithfully represent the Sia-binding properties of corresponding whole organisms, but also demonstrates that the Siglec-like domain-containing SRR adhesins are responsible for the observed binding.

Whole bacteria bind defined sialoglycoproteins in a Sia- and SRR adhesin-dependent manner as well. The evaluation of divalent cation effects on adhesin binding also provides valuable information for understanding differential contribution of bacterial adhesins in different biological niches. In the oral cavity, where free divalent cation concentrations in saliva usually exceed 1 mM in healthy individuals [47], the multiple Ca^2+^/Mg^2+^-dependent adhesins on different bacteria bind their receptors and obscure the Ca^2+^/Mg^2+^-independent Sia-adhesin recognition events. In human blood, the normal plasma level of divalent cations typically exceeds 2 mM [47]. However, the Ca^2+^/Mg^2+^-independent sialic acid-reactive SRR-adhesins become dominant in recognition events in the bloodstream.

Animal models of IE have been used for decades as tools for examining this disease [48]–[50]. However, there are general concerns about merely relying on animal models to infer human relevance [51], [52]. For example, studies of sepsis in mice versus humans have shown markedly different outcomes, which were corroborated by markedly different gene expression profiles [53]. Also, marked differences among human and animal glycomes exist [54], [55]. In this regard, our whole bacterium-whole human blood evaluation is highly complementary to animal studies and most relevant to the native human conditions.

Compared to conventional in vitro studies, several advantages are evident with the WBWB-FC assays developed here. For example, these assays faithfully present all components involved in real human conditions (other than endothelium), a feature incomparable by any other experimental system. Minimal sample manipulation is required. No RBC lysis, wash or separation steps are required, rendering the assays fast and facile. Minimizing manipulations of whole blood not only avoids loss of cells, but also ensures minimal alterations to blood cell phenotypes so that results obtained reflect more accurately the in vivo situations. In addition, the method allows routine analysis using whole blood volumes of merely 10 µL per tube, a considerable reduction compared to standard methods. Finally, the assay is simple, sensitive, and reproducible.

The WBWB-FC assays in the present work provide the first definitive evidence that these oral streptococci can preferentially recognize platelets in intact human whole blood, despite the numerous alternative sialoglycoprotein ligands in plasma and on other blood cells. This preferential platelet-bacterial interaction readily occurs in the fluid whole blood, and may play a significant role in the pathogenesis of IE. Platelets may thus act as discriminative vehicles to deliver particular Sia-binding bacteria to damaged valvular endocardium. In other words, these bacteria inadvertently hitch a ride on circulating platelets and the platelets act as Trojan horse carriers of oral streptococci to the site of damaged endocardium.

Aside from novel mechanistic insights, the present work also provides valuable information from a biomedical point of view. Recent guidelines on the prevention of infective endocarditis have greatly reduced the indications for antimicrobial prophylaxis, in part because of concerns that the risks of these agents may outweigh the benefits [56], [57]. Targeting virulence has been proposed as a promising means to reduce antibiotic use and circumvent increasing antibiotic resistance [58], [59]. The various sialoglycan ligands identified by our glycan microarray screening might be used to produce glycan-based inhibitors against bacterial adhesion, and the rigorously verified Sia-binding adhesin-BR proteins could be potentially developed into prophylactic drugs to block the host target which the opportunistic pathogens exploit to cause IE. As a proof of concept, we have shown that Hsa-BR can successfully block corresponding DL1 binding to platelets in intact human whole blood. Alternatively, these adhesin-BR proteins might be explored as vaccines to boost immunity and generate blocking antibodies against such streptococcal infections. Moreover, the WBWB-FC assay provides a novel method for determination of susceptibility of culprit pathogens to potential virulence inhibitors.

Meanwhile, our data provide an explanation why it is that among numerous microbes that can gain access to the bloodstream, certain viridans group streptococci have a selective advantage in colonizing damaged cardiac valves. Unlike the common situation where pathogens systematically evolve virulence by optimizing binding to specific host targets, this is a case wherein sialoglycan-binding properties that aid normal oral commensalism inadvertently predispose these bacteria to become serendipitous, yet highly specific pathogens.

## Materials and Methods

### Ethics statement

All research involving human participants have been approved by our Institutional Review Board (IRB) or an equivalent committee. Each healthy donor was individually informed and gave his/her written consent for using the collected samples in a scientific study. Collection and use of blood samples included in this study was approved by the Human Research Protections Program (HRPP, #080677X), and saliva samples by the Health Sciences Institutional Review Board (HSIRB, #ORB0511008E).

### Antibodies and other reagents

Allophycocyanine (APC)-conjugated CD41a, fluorescein isothiocyanate (FITC)-conjugated CD62P, and control IgG1-FITC were obtained from Becton Dickinson (San Diego, CA, USA). FITC-conjugated CD235a (Glycophorin A) was obtained from eBioscience (San Diego, CA, USA). Anti-GST SureLight APC was obtained from Columbia Biosciences (Maryland, MD, USA). Alexa Fluor 555-conjugated goat anti-rabbit IgG (H+L) and Glutathione *S*-transferase rabbit IgG antibody fraction were obtained from Molecular Probes (San Diego, CA, USA). Anti-MBP antiserum was obtained from New England Biolabs (San Diego, CA, USA). All chemicals were obtained from Sigma-Aldrich (St. Louis, MO, USA), unless otherwise stated.

### Bacterial strains and plasmids

The bacterial strains and plasmids used in this study are listed in Table S1. Streptococci were grown in Brain Heart Infusion (Becton Dickenson) at 37°C in a 5% CO_2_ environment for 18 h with no agitation. Antibiotic selection was not required for strains carrying chromosomally integrated plasmids or DNA fragments. Strain PS1070 secretes a truncated form of GspB into the culture medium, but has no detectable GspB associated with the cell wall.

### Construction of BR expression plasmids

The *gspB* BR was amplified from chromosomal DNA of *S. gordonii* strains 72-40 and PS478 using primers RGspBgst2 and RGspBgst3, and then cloned in pGEX3X (GE Healthcare) as described for cloning of the BR of strain M99 [60]. The BR coding region of *S. gordonii* strain G9B was similarly amplified from chromosomal DNA using the forward primer 5′AAGGGGATCCCAGAAGCTTCTAGTCAGACAGGCCGG and reverse primer 5′GTGCCTTGCAGAATTCGAAGTACTTCCTTCTCTTGT (BamHI and EcoRI sites are underlined), and then cloned into pGEX3X. Engineered versions of the HsaBR and SrpABR coding regions, with codons optimized for expression in *E. coli*, were subcloned from plasmids pSV278-HsaBR and pSV278-SrpABR, respectively, into pGEX5X.

### Expression and purification of fusion proteins

Cultures of *E. coli* strain BL21 carrying the pGEX expression plasmids were grown in LB with 50 µg/mL carbenicillin until on OD_600_ of approximately 0.6. Cultures were placed on ice for 20 min, and the expression of GST fusion proteins was induced by the addition of IPTG to a final concentration of 0.1 mM. Cultures were incubated for 16 h at 18°C. Cells were harvested by centrifugation and lysed by sonication, and the GST fusion proteins were purified using Glutathione Sepharose 4B (GE Healthcare) according to the manufacturer's instructions. The eluted proteins were exchanged into DPBS and stored at −80°C.

### Fluorescence-labeling of bacteria

Labeling was performed as previously described [61], except that cyanine 3 (Cy3) mono-reactive NHS ester (GE Healthcare) was chosen as the fluorescent label. Cy3-labeled bacteria were washed and resuspended in PBS before use in bacterial overlay, sialoglycan microarray and whole blood binding assays.

### Sialoglycan microarray screening of adhesin-BR proteins and labeled whole bacteria

Glycan microarrays were fabricated using epoxide-derivatized slides as previously described [29]. Printed glycan microarray slides were blocked by ethanolamine, washed and dried, and then fitted in a multi-well microarray hybridization cassette (AHC4X8S, ArrayIt, Sunnyvale, CA, USA) to divide into 8 subarrays. The subarrays were blocked with Ovalbumin (1% w/v) in PBS (pH 7.4) for 1 h at RT, with gentle shaking. Subsequently, the blocking solution was removed by aspiration and diluted adhesin-BR samples were added to each subarray. After incubating the proteins for 2 h at RT with gentling shaking, the slides were extensively washed to remove non-specifically bound proteins. Anti-GST rabbit IgG fraction was added to the subarrays, incubated at RT for 1 h, and washed. Anti-MBP antiserum was used when MBP-tagged Hsa-BR was tested. Fluorescently labeled antibody (Alexa Fluor 555-labeled goat anti-rabbit IgG (H+L), Molecular Probes) was then applied and incubated for 1 h. Following final washes and drying, the developed sialoglycan microarray slides were subjected to scanning by a Genepix 4000B microarray scanner (Molecular Devices Corp., Union City, CA, USA) immediately. For Cy3-labeled whole bacteria, the glycan glass slides were blocked with Ovalbumin (3% w/v) in PBS (pH 7.4) for 1 h at RT, bacteria were subsequently added to the arrays and allowed to interact with the glycans at RT for 2 h. After extensive washing and drying, the bacterium-bound slides were scanned. Data analysis was done using the Genepix Pro 7.0 analysis software (Molecular Devices Corp., Union City, CA). All adhesin-BR fusion proteins and bacterial strains were tested and compared at various concentrations on the arrays. The bacterium-bound glass slides were also imaged by a Keyence microscope (BIOREVO, BZ-9000, Keyence USA).

### Identification of additional Siglec-like BRs

Homologues of the Siglec-like BRs were identified by BLAST search of databases using the GspB signal peptide (residues 1 to 90), a highly conserved region of the *S. gordonii* and *S. sanguinis* SRR glycoproteins.

### Glycan binding to immobilized BR fusion proteins

The binding assay was performed following previously reported protocol [26]. GST-BR fusion proteins were diluted to 0.5 µM in DPBS, and 50 µL applied to microtiter wells. Plates were incubated for 18 h at 4°C, unbound protein was removed by aspiration, and the wells were rinsed three times with DPBS. Multivalent biotinylated carbohydrates (GlycoTech Corporation) were diluted to the indicated concentrations in DPBS containing 1× Blocking Reagent (Roche), 50 µL was added to each well, and the plate was incubated for 3 h at RT with vigorous rocking. Unbound biotinylated carbohydrates were removed by aspiration, and wells were washed three times with 100 µL DPBS. Fifty microliters of streptavidin-conjugated horseradish peroxidase (0.1 µg per mL in DPBS) was added to each well and the plate was incubated for 1 h at RT. The wells were washed twice with 100 µL DPBS, and then 200 µL of a solution of 0.4 mg OPD per mL phosphate-citrate buffer (Sigma) was added to each well. After approximately 15 min, the absorbance at 450 nm was measured.

### Dot blot preparations

Glycoarray dot blots for bacterial overlay were prepared by immobilizing human salivary samples, naturally occurring glycoconjugates (Table S4) and neoglycoconjugates (Table S5) as dots containing 1 µg of protein on nitrocellulose (0.45 µm pore size, Whatman Protran BA 85, Fisher Scientific). The dot blots for examining streptococcal binding to whole plasma were prepared by spotting three-fold serial dilutions of human whole plasma from their original concentrations (∼68 mg/mL) as dots on nitrocellulose. Samples were collected as previously described [62], except that here un-stimulated WS samples were then spun at 12,000× g for 15 min at 4°C to remove debris. The resultant clear supernatant was transferred into a separate polypropylene microtube and stored at −80°C until further use. Samples treated with sialidase from *Clostridium perfringens* (Type X, Sigma-Aldrich) and *Arthrobacter ureafaciens* (ProZyme, Hayward, CA) at a final concentration of 0.05 U/mL at 37°C for 30 min were also included in the dot blots where indicated.

### Bacterial overlay

The overlay method was performed as previously described [61]. In brief, blots were blocked in Tris-buffered saline (0.15 M NaCl, 20 mM Tris HCl and 0.1% NaN_3_) containing 5% BSA (TBS-BSA) for 2 h and then overlaid with labeled bacteria suspended in TBS-BSA (10^8^ bacteria per mL) for 2 h at 4°C in the dark. Next, the blots were washed four times at 4°C for 5 min on a rotary shaker with Tris-buffered saline (0.15 M NaCl, 20 mM Tris HCl and 0.1% NaN_3_) containing 0.05% Tween-20 and dried on gel blot paper (GB003, Whatman). Fluorescence signals of adherent bacteria were detected by a fluorescence scanner (TYPHOON 9400 imaging system, GE Healthcare). For conditions where the overlay was done in the presence of divalent cations, 1 mM CaCl_2_ and 1 mM MgCl_2_ were included in all buffers; while for conditions where the overlay was done in the absence of divalent cations, 5 mM EDTA was included.

### Bacterial-mediated hemagglutination

Blood was collected in Vacutainers (Becton Dickenson) containing 3.2% sodium citrate as anticoagulant. Erythrocytes (RBC) were washed and resusupended in PBS containing 2 mg/mL bovine serum albumin (Sigma-Aldrich, St. Louis, MO) (PBS-BSA) to a final concentration of 10% (v/v). For the removal of sialic acids, RBCs were treated for 1 h at 37°C with sialidase from Clostridium perfringens (Type X, Sigma-Aldrich) and Arthrobacter ureafaciens (ProZyme, Hayward, CA) at final concentration of 0.05 U/mL in PBS. Thereafter, ∼1×10^6^ washed RBCs were used in each well, with two-fold serial dilutions of each bacteria suspension starting from ∼3×10^8^ bacteria per well, in round-bottom wells of a 96-well microtiter plate (Costar, Corning Incorporated, Corning, NY). The endpoints of titrations were determined after overnight incubation at 4°C. Images of hemagglutination were taken with a digital camera (Canon EOS 50D).

### Platelet adherence assay

Bacterial binding to immobilized human platelets was assessed as described [13]. In brief, bacterial strains were grown for 18 h, washed twice with DPBS, sonicated briefly to disrupt aggregated cells, and then diluted to approximately 1×10^7^ bacteria per mL. Fifty microliters of the bacterial suspensions were then applied to microtiter plate wells containing platelet monolayers (∼6×10^6^ platelets) that had been treated 1 h at RT with 100 µL of a blocking solution (1× Blocking Reagent [Roche]) to reduce non-specific adherence. In the case of adhesin-blocking experiment, different concentrations of Hsa-BR were added and incubated for 1 h with the platelet monolayers, prior to the addition of DL1. The plates containing bacteria and platelets were incubated at RT for 3 h with vigorous rocking. Unbound bacteria were then removed by aspiration and washing with DPBS, and the platelet-bound bacteria were released by trypsinization. The number of input and bound organisms was determined by plating serial dilutions of the bacterial suspensions on sheep blood agar, and binding was expressed as the percent of the input bacteria that bound to the platelet monolayers.

### Whole blood flow cytometry assays

Immunolabeling was performed immediately after blood draw. Fresh whole blood was mixed with Cy3-labeled bacteria in different ratios and the mixtures were incubated at room temperature for 0.5 h. Aliquots of the mixtures (10 µL) were added to 5 mL polystyrene round bottom tubes (BD Falcon), followed by adding anti-CD41a-APC and/or anti-CD235a-FITC. After mixing gently, the mixtures were incubated at room temperature for another 0.5 h, and then diluted by PBS (without Ca^2+^/Mg^2+^) to 1 mL total volume for immediate analysis using a FACS Calibur flow cytometer (BD Biosciences). For platelet activation analysis, Cy3-labeled bacteria, anti-CD41a-APC, anti-CD62P-FITC and IgG1-FITC control antibody were used. For adhesin-BR binding, different amounts (6 pmol, 24 pmol, and 96 pmol) of GST-HsaBR were used together with anti-GST-APC. In adhesin inhibition of whole bacterial binding studies, Hsa-BR was added to whole blood samples and the mixtures were incubated at room temperature for 0.5 h before Cy3-labeled bacteria and anti-CD41a-APC were added. The resulting mixtures were incubated at room temperature for another 0.5 h before flow cytometric analysis. All results are expressed as percentage of positive cells, either bacterium-bound or adhesin-bound.

### Statistical analysis

Statistical analyses were performed with GraphPad Prism 6.0 software. Bacterial adhesion to immobilized platelets was statistically analyzed using paired *t* test, bacterial and Hsa-BR binding in whole human blood using 2way ANOVA, and Hsa-BR blocking of DL1 binding in whole blood using 1way ANOVA. *P* values <0.05 were considered statistically significant.

## Supporting Information

Figure S1 **Sialoglycan microarray analysis of binding specificity of MBP-tagged Hsa-BR fusion protein.** The MBP-tagged Hsa-BR show identical Sia-binding specificity as GST-tagged Hsa-BR. (n = 4, SD).
(TIF)

Figure S2 **Imaging of whole bacterial binding on the slide sialoglycan microarrays.** (A) DL1 binding to the sialoglycan microarrays scanned by a Genepix 4000B microarray scanner and visualized using the Genepix Pro 7.0 analysis software. Specific whole bacterial bindings to α2-3-linked Sias #1, 2, and 7-16 are shown. Glycans #3-6, 17, and 18 which are α2-6-linked Sias do not show any binding to DL1. Each glycan was printed on the glass slide in quadruplet. (B and C) Images of different magnifications obtained by a high-resolution Keyence microscope, showing high signal-to-noise ratio whole bacterial binding on the glass slide glycan microarrays.
(TIF)

Figure S3 **Amino acid sequence alignment using ClustalW2.** The BR sequences of the four GspB orthologues were analyzed by ClustalW2. They differ from each other by only a few amino acid residues.
(TIF)

Figure S4 **Sialoglycan microarray analysis of binding specificities of GST-tagged GspB_72-40_BR, GspB_G9B_BR, and GspB_PS478_BR.** (A) Binding of GspB_72-40_BR. (B) Binding of GspB_G9B_BR. (C) Binding of GspB_PS478_BR. (n = 4, SD).
(TIF)

Figure S5 **Overall comparison of bacterial binding to human saliva, purified glycoproteins and related glycoconjugates by dot blot.** Binding of DL1 vs. DL1Δ*hsa*, M99 vs. M99Δ*gspB*, SK36 vs. SK36Δ*srpA* are compared, in the presence or absence of divalent cations.
(TIF)

Figure S6 **Evaluation of platelet activation in whole human blood in the presence of bacteria.** Platelets were gated and examined for activation, as indicated by CD62P expression. RBCs were assessed as an internal negative control from the same whole blood samples.
(TIF)

Figure S7 **Histogram of different concentrations of Hsa-BR binding to platelets in whole blood.**
(TIF)

Figure S8 **Hsa-BR inhibition of DL1 binding to washed and immobilized platelet monolayer.** Different concentrations of Hsa-BR proteins were tested. (n = 3, SEM).
(TIF)

Table S1 **Strains and plasmids employed in the study [63]–[66]** **.**
(DOCX)

Table S2 **Diversity of the sialoglycan library used in the microarrays.**
(DOCX)

Table S3 **Accession numbers of the BR sequences employed in the phylogenetic analysis.**
(DOCX)

Table S4 **Naturally occurring glycoconjugates used in this study [67], [68]** **.**
(DOCX)

Table S5 **Neoglycoproteins used in this study.**
(DOCX)
