# Supplementary figures and images for: Oral Streptococci Utilize a Siglec-Like Domain of Serine-Rich Repeat Adhesins to Preferentially Target Platelet Sialoglycans in Human Blood
Source: PLoS Pathog. 2014 Dec 4;10(12):e1004540. doi: 10.1371/journal.ppat.1004540 (PMC4256463; doi:10.1371/journal.ppat.1004540)

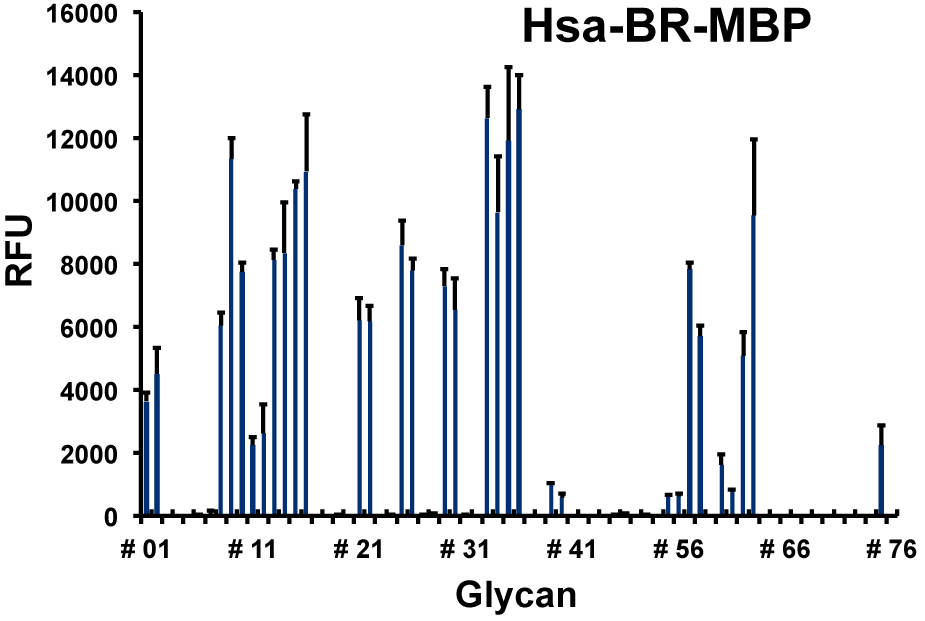

Supplement: Figure S1 — Sialoglycan microarray analysis of binding specificity of MBP-tagged Hsa-BR fusion protein. The MBP-tagged Hsa-BR show identical Sia-binding specificity as GST-tagged Hsa-BR. (n = 4, SD). (TIF) [file ppat.1004540.s001.tif]

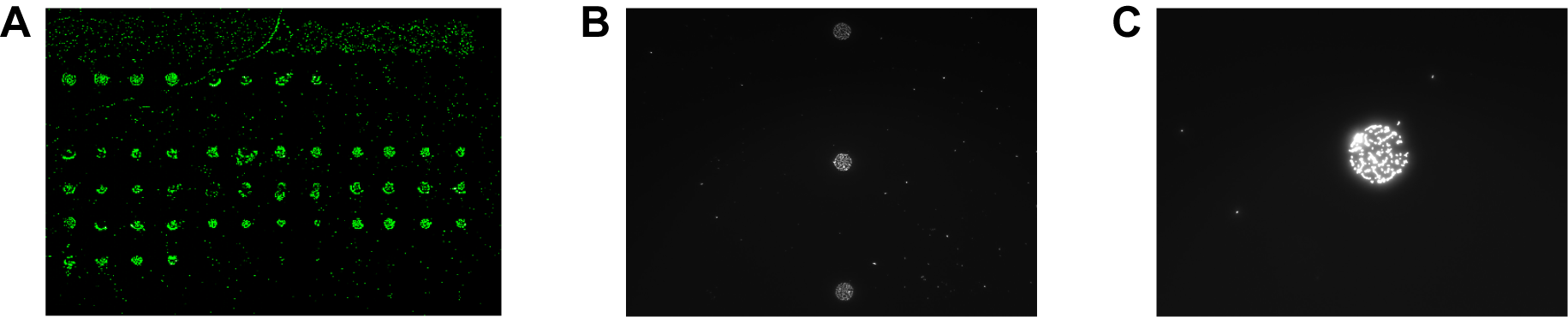

Supplement: Figure S2 — Imaging of whole bacterial binding on the slide sialoglycan microarrays. (A) DL1 binding to the sialoglycan microarrays scanned by a Genepix 4000B microarray scanner and visualized using the Genepix Pro 7.0 analysis software. Specific whole bacterial bindings to α2-3-linked Sias #1, 2, and 7-16 are shown. Glycans #3-6, 17, and 18 which are α2-6-linked Sias do not show any binding to DL1. Each glycan was printed on the glass slide in quadruplet. (B and C) Images of different magnifications obtained by a high-resolution Keyence microscope, showing high signal-to-noise ratio whole bacterial binding on the glass slide glycan microarrays. (TIF) [file ppat.1004540.s002.tif]

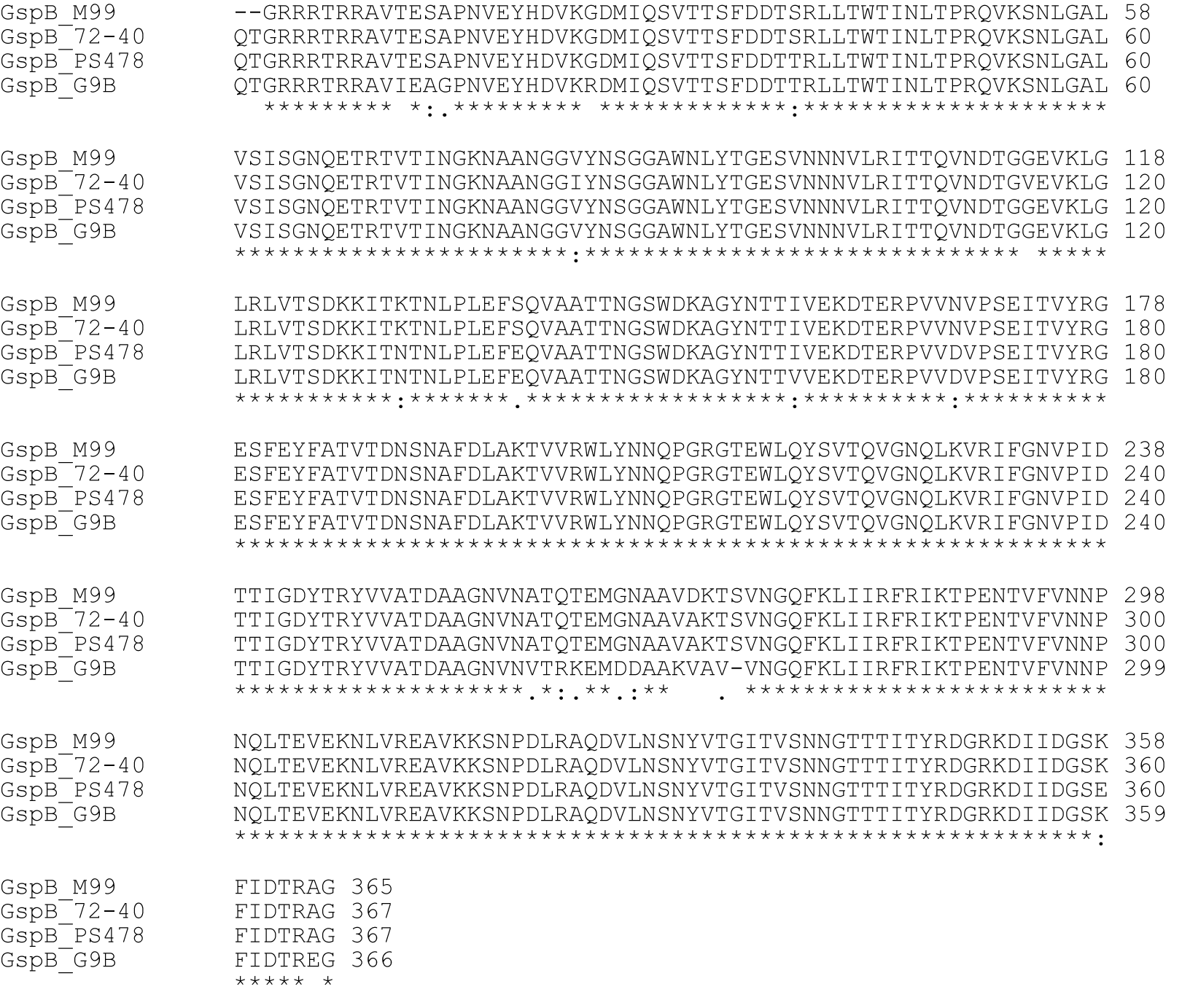

Supplement: Figure S3 — Amino acid sequence alignment using ClustalW2. The BR sequences of the four GspB orthologues were analyzed by ClustalW2. They differ from each other by only a few amino acid residues. (TIF) [file ppat.1004540.s003.tif]

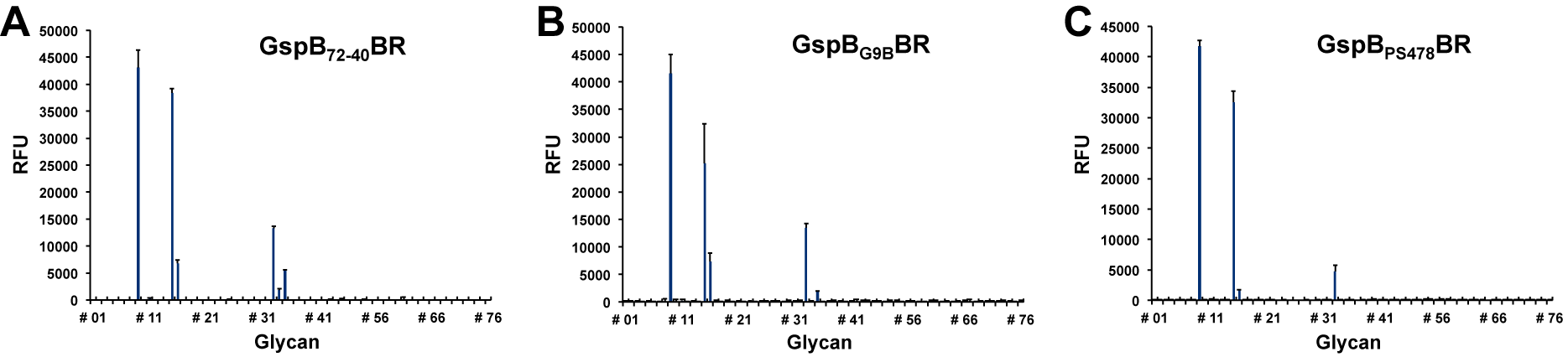

Supplement: Figure S4 — Sialoglycan microarray analysis of binding specificities of GST-tagged GspB72-40BR, GspBG9BBR, and GspBPS478BR. (A) Binding of GspB72-40BR. (B) Binding of GspBG9BBR. (C) Binding of GspBPS478BR. (n = 4, SD). (TIF) [file ppat.1004540.s004.tif]

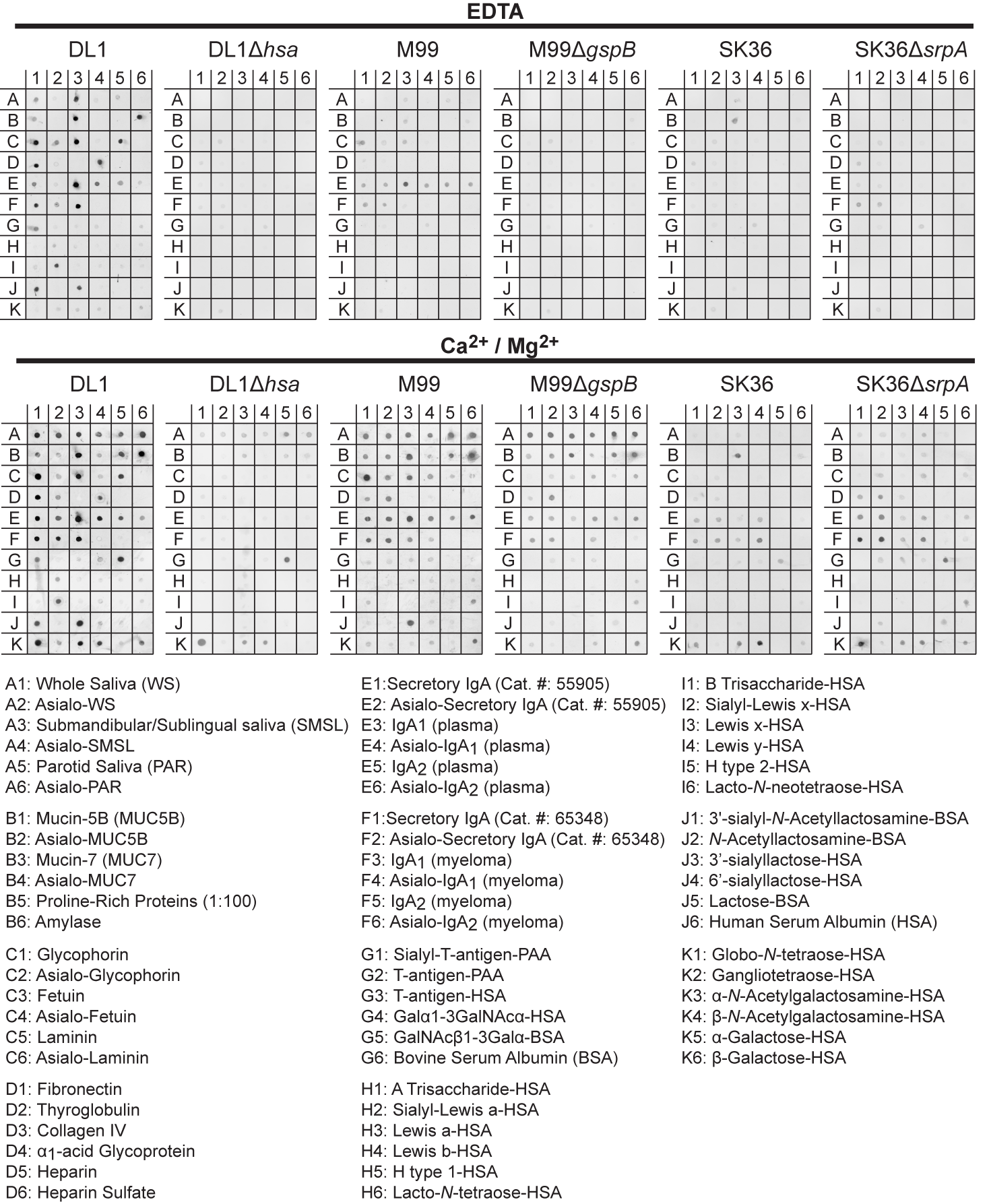

Supplement: Figure S5 — Overall comparison of bacterial binding to human saliva, purified glycoproteins and related glycoconjugates by dot blot. Binding of DL1 vs. DL1Δhsa, M99 vs. M99ΔgspB, SK36 vs. SK36ΔsrpA are compared, in the presence or absence of divalent cations. (TIF) [file ppat.1004540.s005.tif]

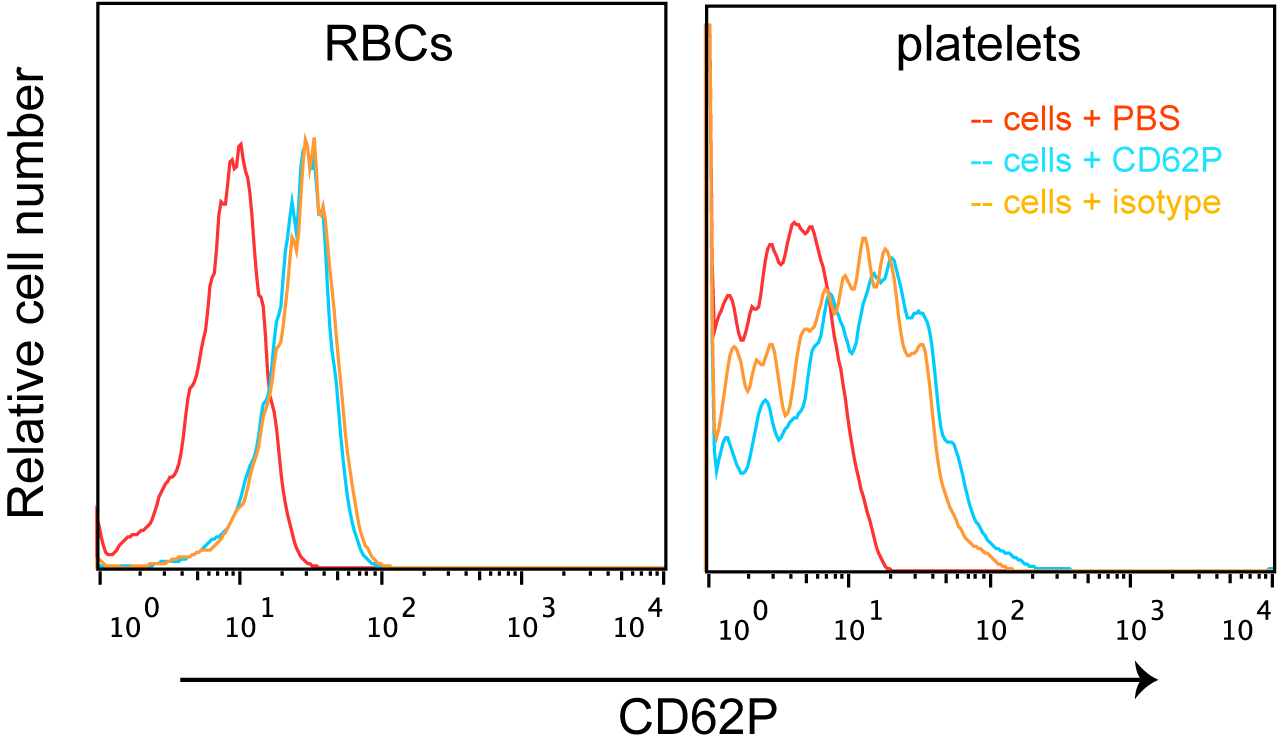

Supplement: Figure S6 — Evaluation of platelet activation in whole human blood in the presence of bacteria. Platelets were gated and examined for activation, as indicated by CD62P expression. RBCs were assessed as an internal negative control from the same whole blood samples. (TIF) [file ppat.1004540.s006.tif]

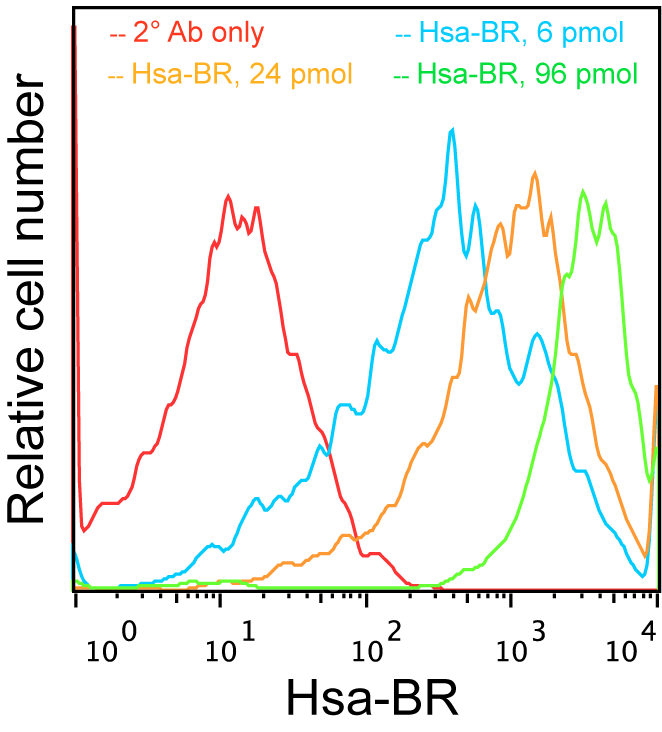

Supplement: Figure S7 — Histogram of different concentrations of Hsa-BR binding to platelets in whole blood. (TIF) [file ppat.1004540.s007.tif]
