# Supplementary material for: Oral Streptococci Utilize a Siglec-Like Domain of Serine-Rich Repeat Adhesins to Preferentially Target Platelet Sialoglycans in Human Blood
Source: PLoS Pathog. 2014 Dec 4;10(12):e1004540. doi: 10.1371/journal.ppat.1004540 (PMC4256463; doi:10.1371/journal.ppat.1004540)
Supplement: Table S1 — Strains and plasmids employed in the study [63]–[66] . (DOCX) [file ppat.1004540.s009.docx]

**Table S1.** Strains and plasmids employed in the study

| **strains/plasmids** | **characteristics** | **source/reference** |
| --- | --- | --- |
| strains |  |  |
| M99 | *S. gordonii* endocarditis isolate | [63] |
| PS846 | M99 ∆*gspB*::pEVP3 | [64] |
| DL1 | *S. gordonii* Challis | [65] |
| UB1545 | DL1 ∆*hsa::aphA3* | [66] |
| SK36 | *S. sanguinis* oral isolate | [15] |
| VT1614 | SK36 *srpA::aphA3* | [15] |
| G9B | *S. gordonii* oral isolate | [32] |
| 72-40^a^ | *S. gordonii* oral isolate | [32] |
| PS1070 | 72-40 *gspB*_1060_::pVA891-3xFLAG | [32] |
| PS478 | *S. gordonii* endocarditis isolate | this study |
|  |  |  |
| plasmids |  |  |
| pGEX-GspBBR | codons 233-615 of GspB | [60] |
| pGEX-HsaBR | codons 228-449 of Hsa | [31] |
| pSV278-HsaBR | 6His-MBP fusion with Hsa codons 228-449^b^ | T. Iverson |
| pSV278-SrpABR | 6His-MBP fusion with SrpA codons 242-453^b^ | T. Iverson |
| pGEX-SrpABR | codons 242-453 of SrpA | this study |
| pGEX-G9BBR | codons 1-366 of G9B | this study |
| pGEX-7240BR | codons 1-367 of 72-40 | this study |
| pGEX-478BR | codons 1-367 of PS478 | this study |

^a^originally identified as *S. sanguinis*, but later typed as *S. gordonii*

^b^optimized for expression in *E. coli*
