# Supplementary material for: Oral Streptococci Utilize a Siglec-Like Domain of Serine-Rich Repeat Adhesins to Preferentially Target Platelet Sialoglycans in Human Blood
Source: PLoS Pathog. 2014 Dec 4;10(12):e1004540. doi: 10.1371/journal.ppat.1004540 (PMC4256463; doi:10.1371/journal.ppat.1004540)
Supplement: Table S2 — Diversity of the sialoglycan library used in the microarrays. (DOCX) [file ppat.1004540.s010.docx]

**Table S2.** Diversity of the sialoglycan library used in the microarrays

| **Saccharide**  **rings** | **Sialic acid**  **types** | **Sialoglycosidic**  **bonds** | **Underlying**  **sugars** |
| --- | --- | --- | --- |
| Monosaccharides | Neu5Ac | α2-3 | Type 1, 2, 3, 4 |
| Disaccharides | Neu5Gc | α2-6 | Core 1 |
| Trisaccharides | Kdn | α2-8 | Lactose |
| Tetrasaccharides | Neu5,9Ac_2_ | α2-3/α2-6 | GalNAc |
| Pentasaccharides | Neu5Gc9Ac | α2-3/α2-8 | Galactose |
|  |  | α2-6/α2-8 | 6S-LacNAc |
|  |  |  | Lewis x |
|  |  |  | 6S-Lewis x |
|  |  |  | LNT |
