# Supplementary material for: Oral Streptococci Utilize a Siglec-Like Domain of Serine-Rich Repeat Adhesins to Preferentially Target Platelet Sialoglycans in Human Blood
Source: PLoS Pathog. 2014 Dec 4;10(12):e1004540. doi: 10.1371/journal.ppat.1004540 (PMC4256463; doi:10.1371/journal.ppat.1004540)
Supplement: Table S3 — Accession numbers of the BR sequences employed in the phylogenetic analysis. (DOCX) [file ppat.1004540.s011.docx]

**Table S3.** Accession numbers of the BR sequences employed in the phylogenetic analysis

| **Name** | **Accession number** |
| --- | --- |
| SK140 | EIF40984 |
| SK355 | AFFN01000016^a^ |
| SK405 | AEWZ01000002^a^ |
| SK678 | EGC27373 |
| DL1 | ABV10391 |
| SK1 | EGF07837 |
| SK1087 | AFDP01000014^a^ |
| SK408 | EGF18650 |
| SK36 | ABN44257 |
| SK1056 | AFFL01000002^a^ |
| AS14 | EJO18696 |
| SK115 | EGD32017 |
| SK150 | EGD35855 |
| SK330 | AFBD01000004^a^ |
| G9B | BAE78662 |
| SK49 | AFFO01000007^a^ |
| PS478 | KM875558 |
| M99 | AAL13053 |
| 72-40 | BAE78661 |
| 2136FAA | ACOI02000004^a^ |

^a^The BR sequence was obtained by independent translation of the deposited DNA sequence
