# Supplementary material for: Oral Streptococci Utilize a Siglec-Like Domain of Serine-Rich Repeat Adhesins to Preferentially Target Platelet Sialoglycans in Human Blood
Source: PLoS Pathog. 2014 Dec 4;10(12):e1004540. doi: 10.1371/journal.ppat.1004540 (PMC4256463; doi:10.1371/journal.ppat.1004540)
Supplement: Table S4 — Naturally occurring glycoconjugates used in this study [67], [68] . (DOCX) [file ppat.1004540.s012.docx]

**Table S4.** Naturally occurring glycoconjugates used in this study

| **Glycoconjugates** | **Origin** |
| --- | --- |
|  |  |
| Salivary mucin-5B (MUC5B) | Prepared as previously described [67]; Gift from Dr. Molakala S. Reddy |
| Salivary mucin-7 (MUC7) | Prepared as previously described [67]; Gift from Dr. Molakala S. Reddy |
| Proline-Rich Proteins (PRP-I) | Prepared as previously described [68]; Gift from Dr. Donald I. Hay |
| Salivary α-amylase | From human saliva; Sigma-Aldrich |
| Glycophorin A | From blood type B-negative; Sigma-Aldrich |
| Fetuin | From fetal calf serum; Sigma-Aldrich |
| Laminin | From human placenta; Sigma-Aldrich |
| Fibronectin | From human plasma; Sigma-Aldrich |
| Thyroglobulin | From bovine thyroid; Sigma-Aldrich |
| Collagen IV | From human placenta; Sigma-Aldrich |
| α_1_-acid Glycoprotein | Human source; Sigma-Aldrich |
| Heparin | From porcine intestinal mucosa; Sigma-Aldrich |
| Heparin sulfate | From bovine kidney; Sigma-Aldrich |
| Secretory IgA (sIgA) | Two samples sIgA that were purified from human colostrum, but by different preparation methods; Cappel, MP Biomedicals; Santa Ana, CA |
| IgA_1_ (plasma) | From human plasma; Athens Research & Technology, Athens, GA |
| IgA_2_ (plasma) | From human plasma; Athens Research & Technology |
| IgA_1_ (myeloma) | From human myeloma plasma; Athens Research & Technology |
| IgA_2_ (myeloma) | From human myeloma plasma; Athens Research & Technology |
