# Supplementary material for: Oral Streptococci Utilize a Siglec-Like Domain of Serine-Rich Repeat Adhesins to Preferentially Target Platelet Sialoglycans in Human Blood
Source: PLoS Pathog. 2014 Dec 4;10(12):e1004540. doi: 10.1371/journal.ppat.1004540 (PMC4256463; doi:10.1371/journal.ppat.1004540)
Supplement: Table S5 — Neoglycoproteins used in this study. (DOCX) [file ppat.1004540.s013.docx]

**Table S5.** Neoglycoproteins used in this study

| **BSA/HSA-glycoconjugates** | **Oligosaccharide conjugates** |
| --- | --- |
| Sialyl-T-antigen-PAA | Neu5Acα2-3Galβ1-3GalNAcα-PAA^a^ |
| T-antigen-PAA | Galβ1-3GalNAcα-PAA^a^ |
| T-antigen-HSA | Galβ1-3GalNAc-APE-HSA^b^ |
| No specified name | (Galα1-3GalNAcα-O-spacer)_n_-HSA^c^ |
| No specified name | (GalNAcβ1-3Galα-O-spacer)_n_-BSA^c^ |
| A Trisaccharide-HSA | GalNAcα1-3[fucα1-2]Galβ1-O-APE-HSA^b^ |
| Sialyl-Lewis a-HSA | Neu5Acα2-3Galβ1-3[Fucα1-4]GlcNAcβ1-3Galβ1-4(Glc)-APD-HSA^b^ |
| Lewis a-HSA | Galβ1-3[fucα1-4]GlcNAcβ1-3Galβ1-4(Glc)-APD-HSA^b^ |
| Lewis b-HSA | Fucα1-2Galβ1-3[Fucα1-4]GlcNAcβ1-3Galβ1-4(Glc)-APD-HSA^b^ |
| H type 1-HSA | Fucα1-2Galβ1-3GlcNAcβ1-3Galβ1-4(Glc)-APD-HSA^b^ |
| Lacto-*N*-tetraose-HSA | Galβ1-3GlcNAcβ1-3Galβ1-4(Glc)-APD-HSA^b^ |
| B Trisaccharide-HSA | Galα1-3[Fucα1-2]Gal-APE-HSA^b^ |
| Sialyl-Lewis x-HSA | Neu5Acα2-3Galβ1-4[Fucα1-3]GlcNAcβ1-3Galβ1-4(Glc)-APD-HSA^b^ |
| Lewis x-HSA | Galβ1-4[Fucα1-3]GlcNAcβ1-3Galβ1-4(Glc)-APD-HSA^b^ |
| Lewis y-HSA | Fucα1-2Galβ1-4[Fucα1-3]GlcNAcβ1-APE-HSA^b^ |
| H type 2-HSA | Fucα1-2Galα1-4GlcNAcβ1-APE-HSA^b^ |
| Lacto-*N*-neotetraose-HSA | Galβ1-4GlcNAcβ1-3Galβ1-4(Glc)-APD-HSA^b^ |
| 3’-sialyl-*N*-acetyllactosamine-BSA | Neu5Acα2-3Galβ1-4GlcNAc-3 atom spacer-BSA^d^ |
| *N*-acetyllactosamine-BSA | Galβ1-4GlcNAc-3 atom spacer-BSA^d^ |
| 3’-sialyllactose-HSA | Neu5Acα2-3Galβ1-4(Glc)-APD-HSA^b^ |
| 6’-sialyllactose-HSA | Neu5Acα2-6Galβ1-4(Glc)-APD-HSA^b^ |
| Lactose-BSA | (Galβ1-4Glcβ-O-spacer)_n_-BSA^c^ |
| Globo-*N*-tetraose-HSA | GalNAcβ1-3Galα1-4Galβ1-4(Glc)-APD-HSA^b^ |
| Gangliotetraose-HSA | Galβ1-3GalNAcβ1-4Galβ1-4(Glc)-APD-HSA^b^ |
| α-*N*-acetylgalactosamine-HSA | GalNAcα1-PAP-HSA^b^ |
| β-*N*-acetylgalactosamine-HSA | GalNAcβ1-PAP-HSA^b^ |
| α-galactose-HSA | Galα1-PAP-HSA^b^ |
| β-galactose-HSA | Galβ1-PAP-HSA^b^ |

^a^GlycoTech, Gaithersburg, MD, United States

^b^IsoSep AB, Tullinge, Sweden

^c^Glycorex AB, Lund, Sweden

^d^Dextra-Laboratories, Reading, United Kingdom
